# miR-155: A Potential Biomarker for Predicting Mortality in COVID-19 Patients

**DOI:** 10.3390/jpm12020324

**Published:** 2022-02-21

**Authors:** Reut Kassif-Lerner, Keren Zloto, Nadav Rubin, Keren Asraf, Ram Doolman, Gidi Paret, Yael Nevo-Caspi

**Affiliations:** 1 Department of Pediatric Critical Care Medicine, Safra Children’s Hospital, Sheba Medical Center, Ramat-Gan 5265601, Israel; reut.kassif@sheba.health.gov.il (R.K.-L.); nadav.rubin@gmail.com (N.R.); gidi.paret@sheba.health.gov.il (G.P.); 2 Department of Obstetrics and Gynecology, Sheba Medical Center, Ramat-Gan 5265601, Israel; kerenzloto@gmail.com; 3 The Dworman Automated-Mega Laboratory, Sheba Medical Center, Ramat-Gan 5265601, Israel; keren.asraf@sheba.health.gov.il (K.A.); ram.doolman@sheba.health.gov.il (R.D.)

**Keywords:** COVID-19, biomarkers, miRNAs, miR-155, predictor of mortality

## Abstract

COVID-19, a pandemic of severe acute respiratory syndrome caused by Coronavirus 2 (SARS-CoV-2), continues to pose diagnostic and therapeutic challenges due to its unpredictable clinical course. Prognostic biomarkers may improve care by enabling quick identification of patients who can be safely discharged home versus those who may need careful respiratory monitoring and support. MicroRNAs (miRNAs) have risen to prominence as biomarkers for many disease states and as tools to assist in medical decisions. In the present study, we aimed to examine circulating miRNAs in hospitalized COVID-19 patients and to explore their potential as biomarkers for disease severity. We studied, by quantitative PCR, the expressions of miR-21, miR-146a, miR-146b, miR-155, and miR-499 in peripheral blood. We found that mild COVID-19 patients had 2.5-fold less circulating miR-155 than healthy people, and patients with a severe COVID-19 disease had 5-fold less circulating miR-155 than healthy people. In addition, we found that miR-155 is a good predictor of COVID-19 mortality. We suggest that examining miR-155 levels in patients’ blood, upon admission to hospital, will ameliorate the care given to COVID-19 patients.

## 1. Introduction

COVID-19, a pandemic of severe acute respiratory syndrome caused by Coronavirus 2 (SARS-CoV-2), continues to pose diagnostic and therapeutic challenges due to its unpredictable clinical course. Approximately a third of COVID-19 patients develop a severe phenotype of the disease that requires intensive care unit (ICU) admission [1]. Optimal medical resource allocation and targeted treatment are essential components of effective care for patients at different stages. These can be achieved following determination of disease progression by subtyping and predicting the outcome of COVID-19 patients. Therefore, there is an urgent need for prognostic biomarkers to aid in the quick identification of patients who can be safely discharged versus those who need careful respiratory monitoring. Such a biomarker would be invaluable when triaging patients on admission to hospital [2].

In recent years, microRNAs (miRNAs) have risen to prominence as a novel tool to assist in medical decisions. These short molecules (~22 nucleotides long) negatively regulate gene expression at the post-transcriptional level by targeting 3′ untranslated regions of complementary specific mRNAs, leading to their degradation or translational repression. miRNAs, which are often tissue-specific, are also present in serum (circulating miRNAs). Such circulating miRNAs can be secreted as a result of passive leakage from cells that suffer injury, chronic inflammation, apoptosis, or necrosis, or they can be actively secreted to exert biological functions in recipient cells to regulate their activity, thereby acting as intercellular signaling molecules [3]. For many disease states, miRNAs have been shown to be sensitive, robust, and cost-effective biomarkers that offer additional information to clinical variables and they are, therefore, already established clinical indicators [4].

In this study, we aimed to examine circulating miR-21, -146a, -146b, -155, and -499 in hospitalized COVID-19 patients. These miRNAs are known to be involved in pathways related to COVID-19. We, therefore, set out to explore their potential as biomarkers for disease severity.

## 2. Materials and Methods

### 2.1. Ethics Statement

The study was performed in full compliance with the Declaration of Helsinki. The protocol was approved by the Institutional Review Board of the Sheba Medical Center (7343-20). This study was performed using residual blood samples under an IRB-approved waiver of informed consent due to it presenting no more than minimal risk to participants.

### 2.2. Patients

This was an observational, single-center study that included 37 patients aged ≥ 18 years, with a positive nasopharyngeal swab PCR test for SARS-CoV-2 during the first and second waves of the pandemic between March 2020 and November 2020. In addition, 15 healthy patients were enrolled as controls. The recruitment process consisted of inviting healthy participants who were interested in taking part in the research. Enrollment criteria included age > 18 years, absence of respiratory symptoms (cough, shortness of breath) or fever, COVID-19 negative status, and informed consent.

The patients were divided according to the severity of their disease at the time of sampling into the following groups: (1) hospitalized COVID-19-positive patients admitted to the general COVID-19 internal medicine wards requiring no respiratory support or oxygen support only i.e., mild group; (2) hospitalized COVID-19-positive patients with respiratory failure requiring invasive or non-invasive ventilation i.e., severe group; and (3) healthy adults. Exclusion criteria included death during one week from admission, extracorporeal support, or hemolytic serum samples.

### 2.3. Data Collection

Comprehensive demographic, clinical, and laboratory data regarding the patients from hospital admission until death or discharge from the hospital were extracted from the electronic medical records (“Cameleon”). Patient vital signs including: temperature, heart rate, respiratory rate, invasive blood pressure, O_2_ saturation, and end-tidal CO_2_, were monitored continuously. Demographic characteristics included age, gender, weight, and BMI. Clinical data included co-morbidities (hypertension, dyslipidemia, diabetes, chronic obstructive pulmonary disease (COPD), malignancy, ischemic heart disease (IHD), chronic renal failure, cerebrovascular accident (CVA)); blood type; hospitalization course; and complications. Laboratory markers were obtained from the patients’ medical records and were performed as part of their routine testing. These included markers of inflammation (C-reactive protein (CRP), TNFα, IL8, IL6, IL-1B); coagulopathy (d-dimer, fibrinogen, INR); acute kidney injury (creatinine); acute cardiac injury (troponin); liver failure (Aspartate aminotransferase (AST), Alanine aminotransferase (ALT), Lactate dehydrogenase (LDH), bilirubin); and routine blood counts (WBC, hemoglobin, platelets). Not all lab tests were performed on all patients. Further data related to the course of hospitalization included length of stay, respiratory support and length of ventilation, as well as mortality.

A widely accepted tool for defining acute lung injury is P/F ratio, which represents the ratio of partial pressure of arterial oxygen levels to the fraction of inspired oxygen (Fio2); however, this requires invasive arterial blood gas sampling. An alternative option is to measure the patient’s saturation over the inspired oxygen (Spo2/Fio2), i.e., S/F ratio, which has been previously validated as a noninvasive criterion for diagnosing lung injury, as the change in Pao2 correlates well with changes in pulse oximetric saturation (Spo2) [5]. Therefore, in this study, we looked at S/F ratio upon presentation to the emergency department, as a screening tool for lung injury, while avoiding blood use, as all patients underwent measurements of Spo2 with documentation of inhaled concentrations of oxygen.

### 2.4. Sample Preparation, RNA Extraction, and cDNA Preparation and Quantification

Sample collection was performed upon admission to the ward/intensive care. Blood samples were collected in ethylenediaminetetraacetic acid (EDTA) tubes and processed within 24 h. The blood was centrifuged at 4 °C for 10 min at 1200× *g* followed by separation of the serum. Centrifugation was then repeated at 4 °C for 10 min at 10,000× *g*. At this stage, samples were frozen at −80 °C until further use. RNA was extracted from 250 μL serum using TRI-Reagent^®^-LS (Sigma, Louis, MO, USA) according to the manufacturer’s protocol. Following the addition of the TRI-Reagent^®^-LS, 5.6 × 10^8^ copies of cel-miR39 were added as an external reference to each sample. Finally, RNA was resuspended in 45 μL H_2_O, of which 5 μL were taken for cDNA synthesis (TaqMan^®^ MicroRNA-RT-Kit, ABI, Thermo Fisher Scientific, Waltham, MA, USA) using a specific primer. Since the overall amount of RNA in plasma is low, the RNA concentration cannot be measured in plasma samples. Therefore, the input RNA amount for analysis is based on the starting value rather than RNA quantity. A consistent input volume was used for all samples. Polymerase chain reaction (PCR) quantification was performed on a StepOne™ machine (Thermo Fisher Scientific, Waltham, MA, USA). Reactions (10 μL) were run in triplicate: 5 μL PCR-mix (ABI); 0.5 μL of either of the Taqman assays (ABI): cel-miR39 (#200), miR-21 (#397), miR-146a (#468), miR-146b (#1097), miR-155 (#2623), and miR-499 (#1352); 2 μL of the cDNA; and 2.5 μL H_2_O. The cycle threshold (Ct) values were calculated with StepOne software v2.3 (Thermo Fisher Scientific, Waltham, MA, USA). ΔCt values were calculated by subtracting the Ct of the selected miRNA from the Ct of cel-miR39 of the same sample. The ΔCt values are inversely correlated with the amount of template miRNA present in the reaction. Comparative quantitation is shown as 2^−ΔCt^.

### 2.5. Statistical Analysis

Categorical variables were described as frequency and percentage. Continuous variables were evaluated for normal distribution using histograms and Q–Q plots. Normally distributed continuous variables were described as mean and standard deviation (SD), and non-normally distributed continuous variables as median and interquartile range (IQR). Correlations between continuous variables were evaluated using the Spearman correlation coefficient. Continuous variables were compared between patients with mild disease versus severe disease, using an independent sample *t*-test or the Mann–Whitney and Kruskal–Wallis tests. Pairwise comparisons significance was assessed by calculating Cohen’s d Effect Size (ES) [6]. Effect sizes (d) above 0.8, between 0.8 and 0.5, between 0.5 and 0.2, and lower than 0.2 were considered as large, moderate, small, and trivial, respectively. The Chi squared or Fisher test was employed to compare categorical variables. Univariate and multivariate logistic regressions were used to evaluate the association between the expression of miRNAs and the severity of the disease. The multivariate model included s/f ratio as confounder. The area under the receiver-operating characteristic (ROC) curve was used to evaluate the discrimination ability of the potential biomarkers (miRNAs). All statistical tests were 2-tailed, and *p* ≤ 0.05 was considered as statistically significant. All calculations were performed using SPSS (Ver. 26.0).

## 3. Results

The expression levels of five miRNAs known to be implicated in molecular pathways linked to COVID-19, were studied in 37 patients with COVID-19 admitted to the COVID-19 clinical wards between March 2020 and November 2020, and in 15 healthy people. The miRNAs studied were miR-21, miR-146a, miR-146b, miR-155, and miR-499. The median age of the participants positive for COVID-19 was 68 (IQR 54.5–79.5) and it was 46 (IQR 42–57) for the healthy participants. A proportion of 26/37 (70%) of the patients with COVID-19 and 4/15 (27%) of the healthy participants were male. Demographic characteristics of the participants are presented in Table 1. The patients were divided into three groups: (1) patients with a mild disease (*n* = 22), (2) patients with a severe disease (*n* = 15) and, (3) healthy adults (*n* = 15).

### 3.1. miR-155 and miR-146b Are Differently Expressed in COVID-19 Patients

**Figure 1:**
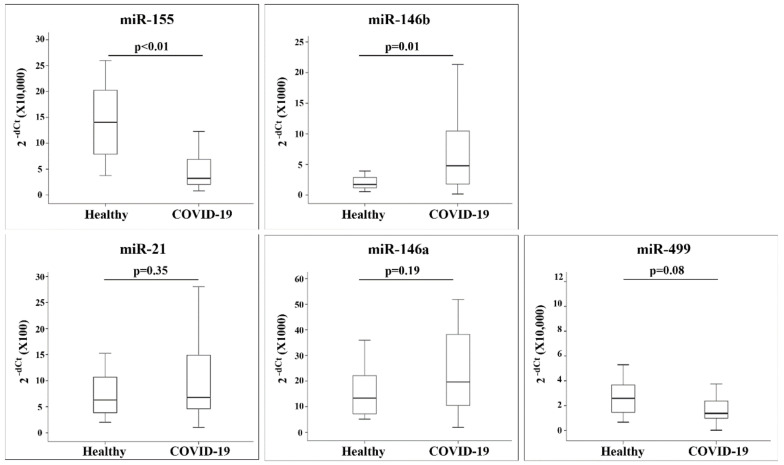
MiRNA expression in healthy and COVID-19 patients: 2^−dCt^ values quantified by quantitative RT-PCR (qRT-PCR) are presented as box plots showing the 10th, 25th, 50th, 75th, and 90th percentile of the patients.

We studied the expression of miRNAs in COVID-19 patients. Samples were obtained upon their admission to the COVID-19 general and critical care wards. In addition, we analyzed miRNAs from healthy participants. The amount of miR-155 and miR-146b differed significantly between the patients with COVID-19 and the healthy participants (*p* ≤ 0.01, for both comparisons): the expression levels of miR-155 were lower in patients with COVID-19 than in the healthy participants, while miR-146b expression was higher in patients with COVID-19 compared with its expression in the healthy participants (Figure 1).

**Figure 2:**
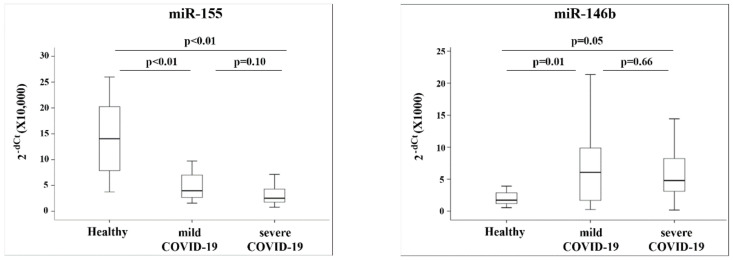
MiRNA expression in healthy, mild and severe COVID-19 patients.

We divided the group of patients with COVID-19 into two sub-groups (as described in the Methods section): patients with mild disease (*n* = 22) and patients with severe disease (*n* = 15). These groups differed in several parameters, including s/f ratio (*p* < 0.001), the number of patients that were intubated (*p* = 0.003), number of days of mechanical ventilation (*p* = 0.006), and the number of hospitalization days (*p* = 0.01) (Table 2). In addition, these two groups differed in several laboratory parameters connected to inflammation and coagulation (Table 2). The expression of the five miRNAs was studied across the three groups: healthy participants, patients with COVID-19 with mild disease, and patients with COVID-19 with severe disease. The amounts of miRs-155 and -146b detected in the patients’ blood differed between the three groups. Specifically, we found a significant difference between the amount of miR-155 in the healthy participants and the group of patients with mild COVID-19, and between the healthy participants and the patients with severe COVID-19 disease (*p* < 0.01 for both comparisons). Calculating the relative amounts of miR-155 in the COVID-19 patients versus the healthy participants revealed that mild patients had 2.5-fold less miR-155 and severe COVID-19 patients had 5-fold less miR-155, when compared with the amount found in the healthy participants. As for miR-146b, the amount differed between the healthy and the mild group (*p* = 0.01) and between the healthy and severe group (*p* = 0.04) (Figure 2). Mild COVID-19 patients had 2.3-fold more miR-146b and severe patients had 1.9-fold more miR-146b than the healthy participants.

2^−dCt^ values quantified by quantitative RT-PCR (qRT-PCR) are presented as box plots showing the 10th, 25th, 50th, 75th, and 90th percentile of the patients.

### 3.2. miR-155 Predicts Patient Outcome

**Figure 3:**
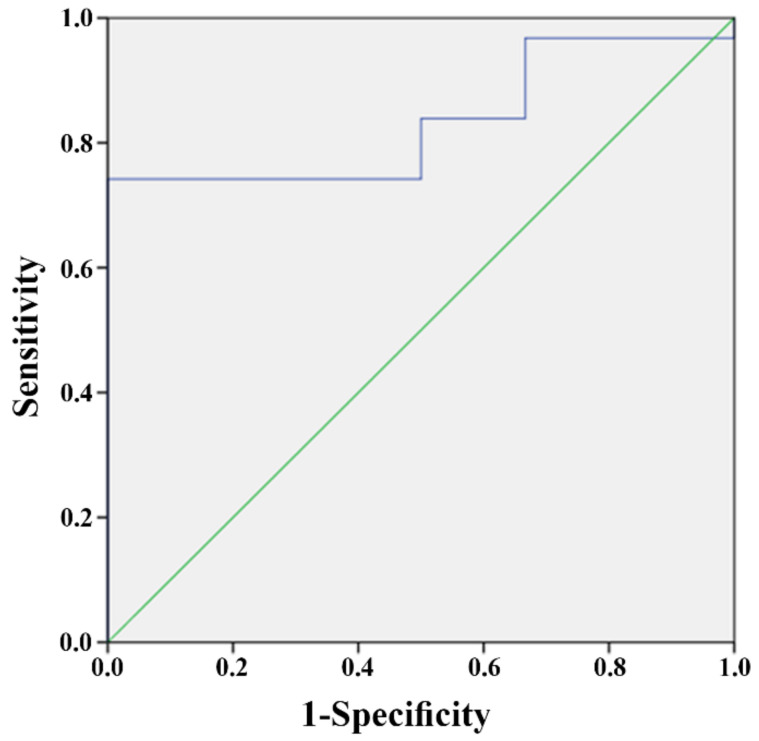
miR-155 can predict a patient’s outcome: ROC curve showing the predictive power of miR-155 levels measured at the time of hospitalization, to predict the patient’s outcome: survival or death (blue line). The green diagonal line represents a random classifier test.

In order to study the predictive value of the miRNAs in the patients’ blood, we divided the patients according to their outcome: patients that died during hospitalization (*n* = 6), and patients that survived (*n* = 31). These two groups did not differ in their demographic parameters (Table S1) but differed in several other characteristics (Table S2): patients that died were ventilated for a longer period of time (*p* = 0.01) and they had longer hospitalization times (*p* = 0.053); all the patients that died were mechanically ventilated, whereas only 38.7% of the patients that survived needed mechanical ventilation (*p* = 0.008). All the deaths in the study were caused by COVID-19 and its complications: two of them (33.4%) occurred within one month from hospitalization. We found that the amount of miR-155, as measured upon hospitalization, was significantly different between these two groups: patients that died had significantly less miR-155 than patients that survived (2^−dCt^ 4.12 vs. 2.07, *p* = 0.01) (effect size = 0.33) (Table S3). The ability of miR-155 to predict whether a patient with COVID-19 would survive was examined using a ROC curve and the area under the curve (AUC). This analysis revealed that miR-155 reached a level of significance, with an AUC of 83.3% (CI 63.7–96.8%; *p* = 0.01) (Figure 3). This analysis also revealed that a 2^−dCt^ value of 1.98 can serve as a cutoff for the prediction of the chances to survive. Specifically, a 2^−dCt^ ≥ than 1.98 predicts that a patient will survive, while a 2^−dCt^ value < than 1.98 predicts that he will not. The sensitivity of this value is 84% and its specificity is 50%.

## 4. Discussion

COVID-19 was declared as a global pandemic on March 2020. Researchers and clinicians alike have been challenged by this outbreak. The clinical manifestations of the disease range from asymptomatic to milder symptoms to more severe and emergent manifestations, such as pneumonia, hypoxemia, and other complications requiring intensive care unit (ICU) admission and mechanical ventilation, at times leading to death. These variations highlighted the need for biomarkers of COVID-19 disease severity to aid in the development of a risk-stratified approach to the care of patients with this illness and have the potential to improve patient outcomes. Immediate categorization of patients into risk groups following diagnosis can ensure optimal resource allocation. Therefore, biomarkers are needed to identify patients who will suffer rapid disease progression to severe complications and death. Given the potential of miRNAs as clinical biomarkers, we analyzed the levels of five miRNAs associated with inflammation and acute lung injury (ALI), key features of COVID-19 [7]. These miRNAs have been shown to regulate the expression of genes related to inflammation: miR-155 has been shown to target SHIP1 and SOCS, two negative regulators of the macrophage inflammatory response [8]; the miR-146 family targets include IRAK1 and TRAF6, involved in regulation of cytokine-responsive gene expression, and the proinflammatory cytokines IL-6, and IL-8 [9]; miR-21 modulates the cytokine IL-12 [10]; and miR-499 targets SOX6, which has been connected to sepsis-induced lung injury [11]. miRNAs analysis was performed on blood samples obtained from COVID-19 patients and healthy participants, upon admission to the hospital, aiming to explore their potential as predictors of mortality.

In the present study, we report two major findings: (1) COVID-19 induces changes in the amounts of circulating miR-155 and miR-146b, and (2) miR-155 is a relevant predictor of mortality from COVID-19 and its complications. To the best of our knowledge, this is the first study to identify a single miRNA as a predictor of mortality in COVID-19 patients. We studied the expressions of five miRNAs (miR-21, -146a, -146b, -155 and -499) in our cohort and found that miRs-146a, -146b, -21 and -499 showed an upward trend in the levels of expression in the sick participants (the results for miR146b are significant). These results are in accordance with the notion that these miRNAs play a role in fighting inflammation or protecting against the damage caused by inflammation [9,10,11,12,13].

As opposed to the results that we obtained for miR-21, -146a, -146b and -499, in our study, miR-155 showed significantly lower levels of expression in the COVID-19 patients. Moreover, we found that the levels of miR-155, as measured upon hospitalization, can predict the patient’s outcome: survival/death. Mir-155 has been shown to be expressed at high levels in ARDS patients [14] and to function as a regulator of antiviral response, including immune and inflammatory responses [15]. Our finding of lower miR-155 levels in COVID-19 patients in comparison with those found in the healthy participants is, therefore, different from what one might expect based on the evidence collected about miR-155’s anti-inflammatory role. There are several possible explanations for this finding: (1) Studies analyzing the roles played by miR-155 were mostly performed in cells where this miRNA exerts its function. In our study, however, we quantified miR-155 in the serum. (2) It is possible that enhanced uptake of miR-155 into the cells occurs in COVID-19 patients and is the cause of this result. (3) It is possible that, upon infection, SARS-CoV-2 enhances selective miRNA degradation. Such a mechanism has been described for Poxvirus, probably in order to promote its replication [16]. To the best of our knowledge, such a mechanism has not yet been described for SARS-CoV-2. Whatever the mechanism causing low levels of circulating miR-155 in COVID-19 patients, the paucity of this miRNA in the serum may contribute to the bad prognosis of COVID-19 patients. Multiple lines of evidence indicate that miRNAs have the potential to be distributed into organs via circulation [17]. Exosome-mediated transfer of miRNAs has been proposed to be a mode of intercellular communication and exosomes have been shown to play a role in inflammation [4,18]. Specifically, miR-155 has been shown to promote the crosstalk between different cells in the tumor microenvironment [19]. We note that lower levels of miR-155 were also found in the serum of patients with coronary artery disease (CAD) when compared with controls [20]. Garg and colleagues studied the levels of several circulating miRNAs in critically ill COVID-19 patients [21]. Their results regarding miR-21 and miR-499 are similar to ours; however, in their study, miR-155 was also expressed at higher levels in the sick patients. The discrepancy between our results and theirs regarding miR-155 may be explained by the fact that all the patients in their first cohort and 90% of those in their second cohort were invasively ventilated and severely ill, whereas in our study, only 47% of the COVID-19 patients were treated with invasive ventilation. In addition, as opposed to the study performed by Garg et al., we omitted from our study samples obtained from patients that were supported by an extracorporeal device, as the centrifuge forces of the pump may cause hemolysis of such samples, which is known to have an impact on extracted cell-free miRNAs [22].

We note that our cohort included a higher percentage of males in the COVID-19 patients versus the healthy participants. Indeed, epidemiological data show a significant sexual dimorphism in COVID-19, with men more infected than women [23]. Several possible explanations have been suggested to explain this phenomenon, including miRNAs that are present on the X-chromosome or estrogen-regulated miRNAs. None of the miRNAs that we studied meets these criteria nor have there been reports on sex-associated differences in their expression. We, therefore, believe that the sex differences between the groups in our cohort did not contribute to our findings regarding the significant differences in miRNA levels. In addition, we are aware that our healthy group of patients was younger than the COVID-19 patients, questioning the effect of age on miRNA expression in our study. Tacke and colleagues found lower miR-155 expression in older patients [24], whereas we found that the oldest group of patients in our study (e.g., mild COVID-19 patients) expressed higher levels of miR-155 than the severe COVID-19 group which was younger. The expressions of miR-146a and -146b were studied by Ong and colleagues, who found that these miRNAs were expressed at higher levels in younger patients [25]. In our study, these miRNAs were expressed at higher levels in the COVID-19 patients who were older than the healthy patients. Hence, we believe that the age difference between our study groups is unlikely to explain the differences in miRNA expression, rather than the differences being a reflection of their disease status.

Several studies have looked for biomarkers indicative of COVID-19 severity. Malik et al. and Ponti et al. found several hematologic and biochemical biomarkers in patient blood tests which are in association with COVID-19 [2,26]. These include lymphopenia, thrombocytopenia, and elevated levels of CRP, procalcitonin, LDH, and D-dimer. Loomba and colleagues found several serum biomarkers, such as white blood cell count, neutrophil count, C-reactive protein, procalcitonin, ferritin, D-dimer, IL-6, and others that are associated with inpatient mortality [27]. In our study, peak WBC, D-Dimer, INR, Fibrinogen, Creatinine, Bilirubin, ALT, LDH, Troponin, and CRP were all in correlation with a more severe illness. However, none of them was able to predict mortality (data not shown).

To date, only a few studies explored circulating miRNAs in COVID-19 patients. These found that SARS-CoV-2 infection induces a robust host miRNA response that could improve COVID-19 detection and patient management [28,29]. De Gonzalo-Calvo and colleagues identified a signature based on two miRNAs (miR-192 and miR-323a) to be a relative predictor of patient outcome in the clinically severe phase of the disease [30]. Our study is the first to identify miR-155 as a single miRNA that can predict mortality upon hospitalization, regardless of the severity of disease at the time of sampling. While host responses to infection are known to be critical in differential outcomes of SARS-CoV-2 infection, the role of miRNAs in COVID-19 pathogenesis is still poorly understood. Targeting of pro-inflammatory miRNAs could present novel therapeutic opportunities against COVD-19, while miRNA profiling may aid in disease detection and surveillance. The use of molecular methods based on miRNAs could refine risk assessment and provide a straightforward approach to guide quick clinical decisions in terms of patient care, monitoring and treatment. Although the suitability as biomarkers for disease is still under research, miRNAs possess an array of advantages to fulfill this role: they are easily accessible, monitored in a cost-efficient protocol, sensitive to a specific medical condition, and tissue-specific [31]. Biomarkers that are able to predict death have been found for several clinical states, such as patients with atrial fibrillation [32] or sepsis [33] and are expected to improve patient care and outcome.

Several limitations should be taken into account when interpreting this study. Although the sample size studied had enough statistical power to detect significance for all the analyses, it would be preferable to include a second independent cohort for validation, with demographic matching of the groups to avoid bias. A large-scale multicenter study is warranted to further validate the performance of miR-155 as a predictor of mortality from COVID-19.

## 5. Conclusions

In conclusion, this study shows that the severity of COVID-19 impacts the profile of circulating miRNAs and that miR-155 can predict patient survival following SARS-CoV-2 infection. Profiling circulating miRNAs emerges as a useful tool for stratification of COVID-19 patients and leads to findings that will ameliorate the care given to COVID-19 patients.

Timely diagnosis and hospitalization, risk stratification, effective utilization of intensive care services, selection of appropriate therapies, monitoring and timely discharge are essential to save the maximum number of lives. Clinical assessment is indispensable, but laboratory markers, or biomarkers, can provide additional, objective information which can significantly impact these components of patient care.

## Figures and Tables

**Table 1:** Demographic and health characteristics of the participants in the study.

Parameter	Healthy (*n* = 15)	COVID-19-Mild (*n* = 22)	COVID-19-Severe (*n* = 15)	*p* Value
Age (years) (Median (IQR))	46 (42–57)	77.5 (62.25–88)	55 (48–66)	<0.001
Gender (male)	4 (27%)	14 (63.63%)	12 (80%)	0.003
BMI	25.0 (21.6–26.8)	26 (25.1–29.33)	31.5 (28.05–38.5)	0.005
Blood type *n* (%)	A+ 7 (47%) B+ 3 (20%) O+ 2 (13%) O− 2 (13%) N/A 1 (7%)	A+ 7 (31%) B+ 1 (4.5%) AB+ 3 (14%) O+ 2 (9%) N/A 9 (41%)	A+ 6 (40%) B+ 3 (20%) AB+ 1 (7%) O+ 3 (20%) N/A 2 (13%)	0.35
Diabetes	1 (6.7%)	8 (36.36%)	1 (6.66%)	0.25
IHD	0 (0)	3 (13.63%)	0 (0%)	0.09
CVE	0 (0)	2 (9.09%)	1 (6.66%)	0.63
Malignancy	0 (0)	1 (4.54%)	1 (6.66%)	0.14
HTN	1 (6.7%)	14 (63.63%)	3 (20%)	0.002
Dyslipidemia	1 (6.7%)	9 (40.9%)	3 (20%)	0.046
CRF	0 (0)	3 (13.63%)	1 (6.66%)	0.43
COPD/CLD	0 (0)	3 (13.63%)	0 (0%)	0.38
Thyroid disease	0 (0)	2 (9.09%)	1 (6.66%)	0.43

Abbreviations: BMI—Body mass index; IHD—ischemic heart disease; CVE—cerebrovascular event; HTN—Hypertension; CRF—Chronic renal failure; COPD—Chronic obstructive pulmonary disease; CLD—chronic lung disease.

**Table 2:** Clinical and laboratory parameters and outcome of patients with COVID-19.

Parameter	Mild (*n* = 22)	Severe (*n* = 15)	*p* Value
WBC peak (K/ μL)	12.5 (10.06–16.27)	17.4 (14.69–24.24)	0.009
HGB nadir (g/dL)	11.84 (8.75–12.48)	7.79 (7.09–10.89)	0.004
D-dimer peak (ng/mL)	1262.5 (650.25–6469)	16,724 (1158–28,955)	0.01
INR peak	1.21 (1.03–1.35)	1.39 (1.24–1.66)	0.005
Fibrinogen peak (mg/dL)	541 (418–760.5)	704 (601–886)	0.04
Creatinine peak (mg/dL)	0.98 (0.78–1.48)	1.18 (1.12–2.89)	0.02
Bilirubin peak (mg/dL)	0.67 (0.51–0.74)	1.28 (1.01–2.68)	0.001
AST-peak (IU/L)	68.41 ± 45.62	810.6 ± 2544.42	0.17
ALT-peak (IU/L)	45.5 (25.5–99.75)	161 (76–231)	<0.001
LDH-peak (IU/L)	445 (333.25–609.75)	540 (515–1038)	0.009
Troponin-peak (ng/L)	17.9 (8.4–28.77)	38.2 (12.9–108)	0.05
CRP-peak (mg/L)	146.5 (65.96–260.25)	300 (234–356)	0.004
IL6 (pg/mL) (*n* = 16)	53 (30.5–218.25)	147.5 (20.75–336)	0.38
IL1B (pg/mL) (*n* = 16)	1 (0.75–1)	0 (0–1)	0.20
IL8 (pg/mL) (*n* = 16)	110 (55.5–255)	66 (44.25–118.25)	0.27
TNFα (pg/mL) (*n* = 16)	32 (23–63)	28.5 (16–42.5)	0.55
s/f ratio upon admission median (IQR)	442.85 (328.68–447.61)	146.66 (108.88–192)	<0.001
Intubated (Yes) *n* (%)	6 (27.27%)	12 (80%)	0.003
Ventilation days *n* (%)	0 (0–11.25)	16 (3–33)	0.006
LOS-Hospitalized days *n* (IQR)	14 (6–28)	32 (21–50)	0.01
Mortality	3 (13.63%)	3 (20%)	0.13
ECMO	0 (0%)	4 (26.67%)	<0.001

Abbreviations: WBC—white blood count; HGB—hemoglobin; INR—international normalized ratio; AST—aspartate aminotransferase; ALT—alanine aminotransferase; LDH—lactate dehydrogenase; CRP—C-reactive protein; TNF—Tumor Necrosis Factor; S/F = Spo2/Fio2; LOS—length of stay; ECMO—extracorporeal membrane oxygenation.

## Data Availability

Not applicable.

## Supplementary Materials

The following supporting information can be downloaded at: https://www.mdpi.com/article/10.3390/jpm12020324/s1, Table S1: Demographic parameters of the COVID-19 patients; Table S2: Clinical parameters and outcome of patients with COVID-19; Table S3: Expression of miRNAs in the blood of patients with COVID-19.

## References

[1] Grasselli G., Greco M., Zanella A., Albano G., Antonelli M., Bellani G., Bonanomi E., Cabrini L., Carlesso E., Castelli G. (2020). Risk Factors Associated With Mortality Among Patients With COVID-19 in Intensive Care Units in Lombardy, Italy. JAMA Intern. Med..

[2] Ponti G., Maccaferri M., Ruini C., Tomasi A., Ozben T. (2020). Biomarkers associated with COVID-19 disease progression. Crit. Rev. Clin. Lab. Sci..

[3] O’Brien J., Hayder H., Zayed Y., Peng C. (2018). Overview of MicroRNA Biogenesis, Mechanisms of Actions, and Circulation. Front. Endocrinol..

[4] Mori M.A., Ludwig R.G., Garcia-Martin R., Brandao B.B., Kahn C.R. (2019). Extracellular miRNAs: From Biomarkers to Mediators of Physiology and Disease. Cell Metab..

[5] Rice T.W., Wheeler A.P., Bernard G.R., Hayden D.L., Schoenfeld D.A., Ware L.B. (2007). National Institutes of Health, N.H.L.; Blood Institute, A.N., Comparison of the SpO2/FIO2 ratio and the PaO2/FIO2 ratio in patients with acute lung injury or ARDS. Chest.

[6] Lakens D. (2013). Calculating and reporting effect sizes to facilitate cumulative science: A practical primer for t-tests and ANOVAs. Front. Psychol..

[7] Wong R.S.Y. (2021). Inflammation in COVID-19: From pathogenesis to treatment. Int. J. Clin. Exp. Pathol..

[8] Mann M., Mehta A., Zhao J.L., Lee K., Marinov G.K., Garcia-Flores Y., Lu L.F., Rudensky A.Y., Baltimore D. (2018). Author Correction: An NF-kappaB-microRNA regulatory network tunes macrophage inflammatory responses. Nat. Commun..

[9] Comer B.S., Camoretti-Mercado B., Kogut P.C., Halayko A.J., Solway J., Gerthoffer W.T. (2014). MicroRNA-146a and microRNA-146b expression and anti-inflammatory function in human airway smooth muscle. Am. J. Physiol. Lung Cell. Mol. Physiol..

[10] Lu T.X., Munitz A., Rothenberg M.E. (2009). MicroRNA-21 is up-regulated in allergic airway inflammation and regulates IL-12p35 expression. J. Immunol..

[11] Zhang W., Li J., Yao H., Li T. (2021). Restoring microRNA-499-5p Protects Sepsis-Induced Lung Injury Mice Via Targeting Sox6. Nanoscale Res. Lett..

[12] Li L., Chen X.P., Li Y.J. (2010). MicroRNA-146a and human disease. Scand. J. Immunol..

[13] Zhang C., Wang H., Yang B. (2020). miR-146a regulates inflammation and development in patients with abdominal aortic aneurysms by targeting CARD10. Int. Angiol. A J. Int. Union Angiol..

[14] Wu X., Wu C., Gu W., Ji H., Zhu L. (2019). Serum Exosomal MicroRNAs Predict Acute Respiratory Distress Syndrome Events in Patients with Severe Community-Acquired Pneumonia. BioMed Res. Int..

[15] Lu L.F., Gasteiger G., Yu I.S., Chaudhry A., Hsin J.P., Lu Y., Bos P.D., Lin L.L., Zawislak C.L., Cho S. (2015). A Single miRNA-mRNA Interaction Affects the Immune Response in a Context- and Cell-Type-Specific Manner. Immunity.

[16] Backes S., Shapiro J.S., Sabin L.R., Pham A.M., Reyes I., Moss B., Cherry S., tenOever B.R. (2012). Degradation of host microRNAs by poxvirus poly(A) polymerase reveals terminal RNA methylation as a protective antiviral mechanism. Cell Host Microbe.

[17] Mittelbrunn M., Gutierrez-Vazquez C., Villarroya-Beltri C., Gonzalez S., Sanchez-Cabo F., Gonzalez M.A., Bernad A., Sanchez-Madrid F. (2011). Unidirectional transfer of microRNA-loaded exosomes from T cells to antigen-presenting cells. Nat. Commun..

[18] Bala S., Csak T., Momen-Heravi F., Lippai D., Kodys K., Catalano D., Satishchandran A., Ambros V., Szabo G. (2015). Biodistribution and function of extracellular miRNA-155 in mice. Sci. Rep..

[19] Zheng Z., Sun R., Zhao H.J., Fu D., Zhong H.J., Weng X.Q., Qu B., Zhao Y., Wang L., Zhao W.L. (2019). MiR155 sensitized B-lymphoma cells to anti-PD-L1 antibody via PD-1/PD-L1-mediated lymphoma cell interaction with CD8+T cells. Mol. Cancer.

[20] Faccini J., Ruidavets J.B., Cordelier P., Martins F., Maoret J.J., Bongard V., Ferrieres J., Roncalli J., Elbaz M., Vindis C. (2017). Circulating miR-155, miR-145 and let-7c as diagnostic biomarkers of the coronary artery disease. Sci. Rep..

[21] Garg A., Seeliger B., Derda A.A., Xiao K., Gietz A., Scherf K., Sonnenschein K., Pink I., Hoeper M.M., Welte T. (2021). Circulating cardiovascular microRNAs in critically ill COVID-19 patients. Eur. J. Heart Fail..

[22] Kirschner M.B., Edelman J.J., Kao S.C., Vallely M.P., van Zandwijk N., Reid G. (2013). The Impact of Hemolysis on Cell-Free microRNA Biomarkers. Front. Genet..

[23] Pontecorvi G., Bellenghi M., Ortona E., Care A. (2020). microRNAs as new possible actors in gender disparities of COVID-19 pandemic. Acta Physiol..

[24] Tacke F., Spehlmann M.E., Vucur M., Benz F., Luedde M., Cardenas D.V., Roy S., Loosen S., Hippe H.J., Frey N. (2019). miR-155 Predicts Long-Term Mortality in Critically Ill Patients Younger than 65 Years. Mediat. Inflamm..

[25] Ong J., Woldhuis R.R., Boudewijn I.M., van den Berg A., Kluiver J., Kok K., Terpstra M.M., Guryev V., de Vries M., Vermeulen C.J. (2019). Age-related gene and miRNA expression changes in airways of healthy individuals. Sci. Rep..

[26] Malik P., Patel U., Mehta D., Patel N., Kelkar R., Akrmah M., Gabrilove J.L., Sacks H. (2021). Biomarkers and outcomes of COVID-19 hospitalisations: Systematic review and meta-analysis. BMJ Evid.-Based Med..

[27] Loomba R.S., Villarreal E.G., Farias J.S., Aggarwal G., Aggarwal S., Flores S. (2021). Serum biomarkers for prediction of mortality in patients with COVID-19. Ann. Clin. Biochem..

[28] Farr R.J., Rootes C.L., Rowntree L.C., Nguyen T.H.O., Hensen L., Kedzierski L., Cheng A.C., Kedzierska K., Au G.G., Marsh G.A. (2021). Altered microRNA expression in COVID-19 patients enables identification of SARS-CoV-2 infection. PLoS Pathog..

[29] Li C., Hu X., Li L., Li J.H. (2020). Differential microRNA expression in the peripheral blood from human patients with COVID-19. J. Clin. Lab. Anal..

[30] de Gonzalo-Calvo D., Benitez I.D., Pinilla L., Carratala A., Moncusi-Moix A., Gort-Paniello C., Molinero M., Gonzalez J., Torres G., Bernal M. (2021). Circulating microRNA profiles predict the severity of COVID-19 in hospitalized patients. Transl. Res. J. Lab. Clin. Med..

[31] Condrat C.E., Thompson D.C., Barbu M.G., Bugnar O.L., Boboc A., Cretoiu D., Suciu N., Cretoiu S.M., Voinea S.C. (2020). miRNAs as Biomarkers in Disease: Latest Findings Regarding Their Role in Diagnosis and Prognosis. Cells.

[32] Sharma A., Hijazi Z., Andersson U., Al-Khatib S.M., Lopes R.D., Alexander J.H., Held C., Hylek E.M., Leonardi S., Hanna M. (2018). Use of Biomarkers to Predict Specific Causes of Death in Patients With Atrial Fibrillation. Circulation.

[33] Pregernig A., Muller M., Held U., Beck-Schimmer B. (2019). Prediction of mortality in adult patients with sepsis using six biomarkers: A systematic review and meta-analysis. Ann. Intensive Care.

