# Supplementary material for: miR-155: A Potential Biomarker for Predicting Mortality in COVID-19 Patients
Source: J Pers Med. 2022 Feb 21;12(2):324. doi: 10.3390/jpm12020324 (PMC8877479; doi:10.3390/jpm12020324)
Supplement: Supplementary file 1 [file jpm-12-00324-s001.zip › jpm-1582068-supplementary.pdf]

Table S1: Demographic parameters of the COVID-19 patients

Parameter	Survivors (n=31)	Dead (n=6)	*P* value
Age (years) (Median (IQR))	69(54-81)	63(53.75-77.5)	0.65
Gender (male)	21(67.74%)	5(83.33%)	0.64
Blood type	A+ 9(47.36%)	A+ 3(50%)	0.72
	B+ 4(21.05%)	AB+ 1(16.7%)	
	AB+ 3(15.78%)	O+ 2(33.3%)	
	O+ 3(15.78%)		
	N/A 12		
BMI	27.75(25.2-32.43)	30.9(23.1-35)	0.90
Diabetes	7(22.58%)	2(33.33%)	0.62
IHD	3(9.67%)	0(0%)	1.00
CVE	2(6.45%)	1(16.66%)	0.42
Malignancy	2(6.45%)	0(0%)	1.00
HTN	14(45.16%)	3(50%)	1.00
Dyslipidemia	9(29.03%)	3(50%)	0.4
CRF	4(12.9%)	0(0%)	1.00
COPD/CLD	2(6.45%)	1(16.66%)	0.42

Abbreviations: BMI- Body mass index; IHD-ischemic heart disease; CVE-cerebrovascular event; HTN-hypertension; CRF-chronic renal failure; COPD-chronic obstructive pulmonary disease; CLD-chronic lung disease

Table S2: Clinical parameters and outcome of patients with COVID-19

Parameter	Home /rehab (n=31)	Death (n=6)	*P* value
s\f ratio	320(125-442.85)	234.8(144.28-373.8)	0.95
Ventilation days	0(0-16)	21(12-45)	0.01
Hospitalized days	15(8-33)	31(26.75-64.5)	0.05
Intubated	12(38.7%)	6(100%)	0.008
Worst respiratory support:			
No respiratory support	15(48.39%)	0	0.10
With respiratory support (non-invasive or invasive)	13(41.94%)	5(83.33%)	
ECMO	3(9.67%)	1(16.67%)	0.52

Abbreviations: S/F = Spo2/Fio2; ECMO – extracorporeal membrane oxygenation

Table S3: Expression of miRNAs in the blood of patients with COVID-19

MiRNA	Survivors (n=31)	Dead (n=6)	*P* value
MiR-21 (2^{-dCt} X 100)	7.04(4.51-13.76)	7.93(4.91-24.3)	0.56
MiR-146a (2^{-dCt} X 1000)	18.57(9.68-35.66)	22.41(7.09-58.45)	0.96
Mi146b (2^{-dCt} X 1000)	5.27(3.07-9.85)	4.9(1.3-13.61)	1.00
MiR-155 (2^{-dCt} X 10000)	4.12(2.08-7.01)	2.07(1.34-2.56)	0.01
MiR-499 (2^{-dCt} X 1000)	1.4(1.17-2.41)	0.98(0.35-1.77)	0.11
